# Quantification of effectiveness prior to clinical application: the example of silicone socks for diabetics

**DOI:** 10.1186/1757-1146-5-S1-P9

**Published:** 2012-04-10

**Authors:** Claudia Giacomozzi, Jessica Crafoord, Emanuela D’Ambrogi, Luigi Uccioli

**Affiliations:** 1 Dept. of Technology and Health, Italian National Institute of Health (ISS), Rome, Italy; 2 Southern Älvsborg Hospital, Borås, Sweden; 3 Dept. of Internal Medicine, University of Rome “Tor Vergata”, Italy

## Background

To avoid abnormal foot local loading while maintaining a physiological foot biomechanics does represent a challenge in the management of Diabetic foot and a valuable instrument to counter the onset of the ulceration process. The authors hypothesized that silicone socks may help in keeping dynamic peak pressures below risky values. The study describes the methodology the authors used to assess the effectiveness of the socks prior to the launch of the clinical application on Diabetic patients.

## Materials and methods

Silicone socks were tested on a healthy volunteer during barefoot walking without and with socks; a calibrated EMED pressure platform – capacitive technology, 4sens/cm^2^, accuracy 3% - was used at 50Hz to acquire 30 at regimen steps for the left foot under each condition; data were temporally normalised and averaged; main kinetic parameters were extracted; vertical force values and curves were used to characterize the volunteer’s foot biomechanics.

## Results

Student’s t-test delivered p values <0.05 for duration and for the 3 relevant values of the force curve (load acceptance peak, midstance valley, propulsion peak). Invariance of force curve was also verified through the linear regression test (r^2^ = 0.996; slope = 1.03) and the paired t-test (p=0.543). Finally, the %RMSE calculated over the entire force curve was equal to 2%. On the contrary, the analysis of peak pressure curve and values showed that: i) curves were quite similar in shape but different in values especially from load transfer to propulsion (r^2^ = 0.929; slope = 0.70; %RMSE = 13%); ii) averaged peak pressure reduction was 165kPa, i.e. about 30% of the barefoot value and 13% of the device full scale (1270kPa).

**Figure 1:**
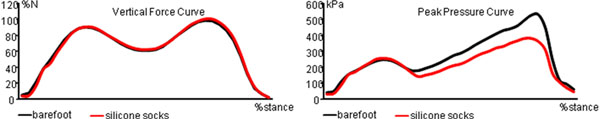
Vertical Force and Peak Pressure curves obtained by barefoot walking without (black) and with (red) silicone socks (curves have been averaged over 30 left steps).

## Conclusions

Difference in force <3% - the device accuracy level - and difference in peak pressure and in %RMSE much > 3% proved the potential effectiveness of the silicone socks in reducing local loading without changing foot biomechanics. Tests under different walking speeds, body weights and peak pressures are in progress.

